# Intraoperative Diagnosis and Surgical Procedure with Imprint Cytology for Small Pulmonary Adenocarcinoma

**DOI:** 10.7150/jca.35026

**Published:** 2020-02-20

**Authors:** Tomoyuki Nakagiri, Tomio Nakayama, Toshiteru Tokunaga, Akemi Takenaka, Hidenori Kunoh, Hiroto Ishida, Yasuhiko Tomita, Shin-ichi Nakatsuka, Harumi Nakamura, Jiro Okami, Masahiko Higashiyama

**Affiliations:** 1 Department of General Thoracic Surgery, Osaka International Cancer Institute, Osaka, Japan; 2 Cancer Control and Statistics, Osaka International Cancer Institute Osaka, Japan; 3 Department of Cytology, Osaka International Cancer Institute, Osaka, Japan; 4 Department of Pathology, Osaka International Cancer Institute, Osaka, Japan

**Keywords:** small size lung adenocarcinoma, intraoperative diagnosis, surgical procedure, outcome, Nakayama-Higashiyama's classification

## Abstract

**Objectives**: For patients with multiple small-sized pulmonary cancers, a lobectomy can disrupt future therapeutic options for other lesions. It was recently reported that limited pulmonary resections were not inferior to lobectomy for the management of selected peripheral small-sized pulmonary adenocarcinomas. Patients with adenocarcinoma *in situ* or minimally invasive adenocarcinoma, as proposed by the International Association for the Study of Cancer classification, have been reported to have 100% survival after 5 years. However, that classification can be applied postoperatively. Since 2005, we have been intentionally performing limited pulmonary resection procedures for small-sized adenocarcinoma cases based on intraoperative imprint cytological diagnosis and our classification (Nakayama-Higashiyama's classification).

**Materials and Methods**: A total of 120 consecutive cases were included in this study. Lung tumors were removed intraoperatively by wedge resection, and stump smear cytology was performed, from which the cases were classified into 5 groups based on our classification. When the tumor was classified as Group I or II, the operation was finished. When diagnosed as a more advanced classification, a lobectomy and lymph node dissection were additionally performed.

**Results**: The 5-year survival rate for Group I and II was 100%, while those for Group III and IV-V were 95.8% and 94.4%, respectively. The 5-year disease-free survival rates for Group I and Group II were 100% and 97.1%, respectively, and for Group III and IV-V they were 100% and 94.1%, respectively.

**Conclusion**: Use of cytological findings along with Nakayama-Higashiyama's classification for determining operation procedure is effective for treatment of patients with small-sized pulmonary adenocarcinoma.

## Introduction

With the improved resolution of computer tomography (CT) and/or spread of CT screening, we often find small multiple ground-glass opacities (GGOs) in lesions in the lungs. It is often difficult to diagnose these lesions with bronchoscopy alone. Intraoperative diagnosis is often needed for these lesions. In addition, the incidence of second primary lung cancer after resection of non-small cell lung cancer (NSCLC) has been estimated to be 1% to 4% per patient-year,^1^-^4^ and even after resection of stage I NSCLC, the incidence was reported to be 2% per patient-year.^4^ In such cases, a lobectomy can disturb future therapeutic options for other lesions or second primary lesions.

It was recently reported that a limited pulmonary resection procedure, including a wedge resection and segmentectomy, was not inferior to lobectomy for the management of peripheral small-sized pulmonary adenocarcinomas, especially adenocarcinoma* in situ* (AIS) or minimally invasive adenocarcinoma (MIA) in the International Association for the Study of Cancer (IASLC)/American Thoracic Society (ATS)/European Respiratory Society (ERS) classification of lung adenocarcinoma.^5^,^6^ The survival of patients with AIS or MIA is 100% at 5 years after the operation.^7^ In the early stage of such types of lung cancers, it is better to resect the lesion with wedge resection, which needs limited hilar vessel handling compared to segmentectomy, considering the potential need for future surgery. To decide whether to perform a limited pulmonary resection or lobectomy, a method of intraoperative diagnosis is needed.

For conventional intraoperative diagnosis, a biopsy, especially partial resection of the tumor, is needed, despite the small size of the tumor. However, according to the classification of the IASLC 8^th^ TNM system, we have to describe the tumor by size, invasion of peripheral structures, and as adenocarcinoma, type of adenocarcinoma subclassification as the postoperative diagnosis. With the conventional intraoperative biopsy (or partial resection) for cryosection, part of the tumor is lacking. As a result, one cannot accurately diagnose the tumor pathologically after the operation. To diagnose the tumor accurately, we have to conserve the tumor, as much as possible; thus, another intraoperative diagnosis method is needed.

Previously, we reported a cytological method, termed Nakayama-Higashiyama's classification (N-H classification), in which imprint cytology findings of small adenocarcinomas are correlated with the IASLC adenocarcinoma pattern classification, WHO 4^th^ adenocarcinoma histological grading, and the 8^th^ IASLC TNM classification.^8^ Although other studies have also reported the usefulness of cytological smear findings for small-sized pulmonary adenocarcinoma cases,^9^,^10^ there are no reports of its clinical application. Since August 2005, we have been intentionally performing limited pulmonary resection procedures for small-sized adenocarcinoma cases based on intraoperative imprint cytological diagnosis and the N-H classification. The results of a comparison between the classification and GGO rates, and a prospective study of our procedure selection are presented.

## Materials and Methods

### Sample collection

The lung tumors in the present patients were resected by wedge resection. The samples were cut at the center of the tumor and smeared onto microscope slides. The preparations were immediately fixed in the operation room and evaluated (Supplement Fig. 1^8^).

### Classification

Since 1993, we have been performing imprint cytology intraoperatively and classifying the samples into 5 groups based on the N-H classification (Table 1, Supplement Fig. 2^8^), as recently reported.^8^ For the present study, adenocarcinomas smaller than 2 cm, excluding mucinous adenocarcinoma cases, were examined.

### Patients

Since August 2005, we have been performing limited surgery with intraoperative imprint cytology based on the N-H classification. The present study included consecutive patients with small lung adenocarcinoma (≤1.5 cm) or GGO-predominant small adenocarcinoma (≤2.0 cm) tumors. Patients with clinical T1a-bN0 lung adenocarcinomas according to the 8^th^ edition and who underwent surgery from August 2005 to February 2010 and subsequent follow-up were analyzed. Written, informed consent was obtained from all patients, and the ethical review board of our institution approved this study (Approval No. 18055).

### Operation procedure selection

**Figure 1:**
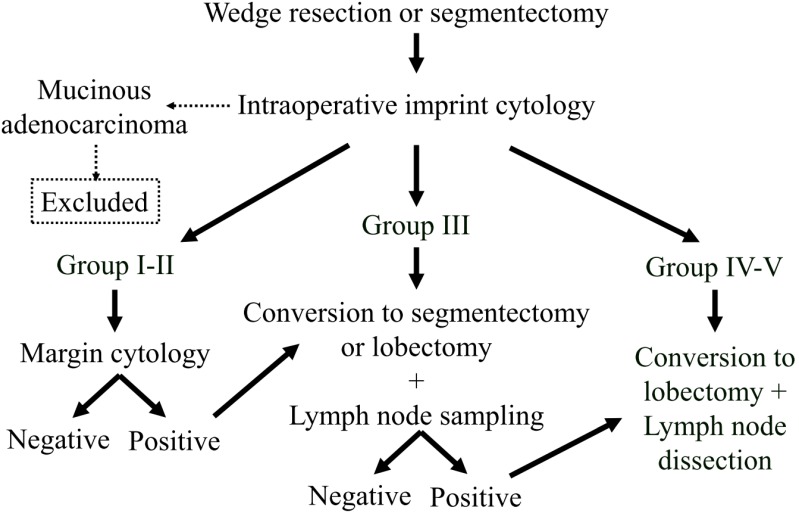
Algorithm for selection of operation extent. All lesions aere resected as part of a limited resection procedure and diagnosed with our cytology method. When the tumor is a mucinous adenocarcinoma, the patient is removed from the analysis. When the tumor is diagnosed as Group I or II and the margin cytology is negative, the operation is finished. When the tumor is diagnosed as Group III, the operation is converted to a segmentectomy or lobectomy, and lymph node sampling is performed. In addition, when a lymph node is diagnosed as positive, the segmentectomy is converted to a lobectomy with lymph node dissection. When the tumor is diagnosed as Group IV or V, the operation is converted to a lobectomy with lymph node dissection.

The algorithm for deciding operation extent is shown in Figure 1. Choice of a limited resection, wedge resection, or segmentectomy was determined according to tumor location, and then the procedure was planned and performed for all patients. Imprint cytology, N-H classification, and margin cytology procedures were performed on an intraoperative basis. In cases of mucinous adenocarcinoma, the patient was excluded from the analysis. When the tumors were classified as N-H Group I or II and the margin cytology was negative, the operation was finished at that point. In patients with tumors classified as N-H Group III, conversion to a segmentectomy or lobectomy with lymph node sampling was done. In addition, after converting to segmentectomy, when any lymph node was diagnosed as positive, we converted to lobectomy along with mediastinal lymph node dissection. In cases classified as N-H Group IV or V, we converted to a standard operation including lobectomy and mediastinal lymph node dissection.

### Statistical analysis

Differences in incidence between 2 or more groups were compared using contingency table analysis with Pearson's χ^2^ test. Kaplan-Meier analysis and the log-rank test were used for survival curves and their comparisons, respectively. Values of *p*≤0.05 were considered significant.

## Results

### Patients

Of the 127 patients, 7 had mucinous adenocarcinomas; thus, a total of 120 patients were analyzed (Table 2). Fifty patients were male (41.7%), and the mean age was 61.6±10.1 years. Mean tumor size was 13.1±4.1 mm, and the GGO rate was 70.8%±30.0%. According to the N-H classification, 38 patients were Group I, 40 Group II, 24 Group III, 17 Group IV, and 1 Group V. A wedge resection was performed for 67 patients, and a segmentectomy was performed for 31. Furthermore, a lobectomy was performed for 22 patients, including 1 in Group II because of positive margin cytology findings and 7 in Group III.

### Tumor size, GGO rate, and lymphatic and vessel involvement

**Figure 2:**
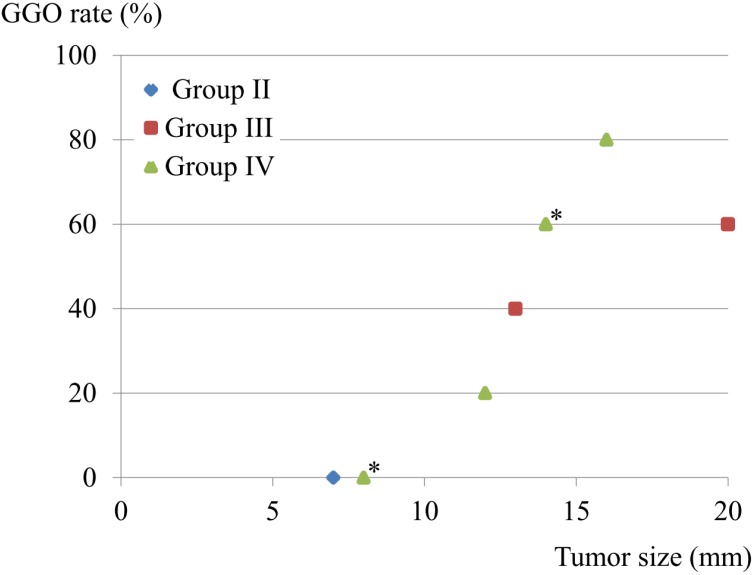
Tumor size, GGO rate, and lymphatic involvement. Nine patients had lymphatic involvement, and 3 had both lymphatic and vessel involvement, while none had a lesion with only vessel involvement. GGO rate and tumor size were compared in the 9 patients with lymphatic involvement. One patient classified as Group II had a 7-mm solid component. *Two cases overlapped.

There were 9 patients with lymphatic involvement, of whom 3 showed involvement of both lymph nodes and vessels, while none had vessel involvement alone. The GGO rate and tumor size were compared in these 9 patients. One Group II patient had a 7-mm solid component. Interestingly, patients in Group III-IV with GGO-predominant lesions also showed lymphatic involvement, though that was avoided by the limited operation (Fig. 2).

### Outcomes after operation

#### Overall survival

**Figure 3:**
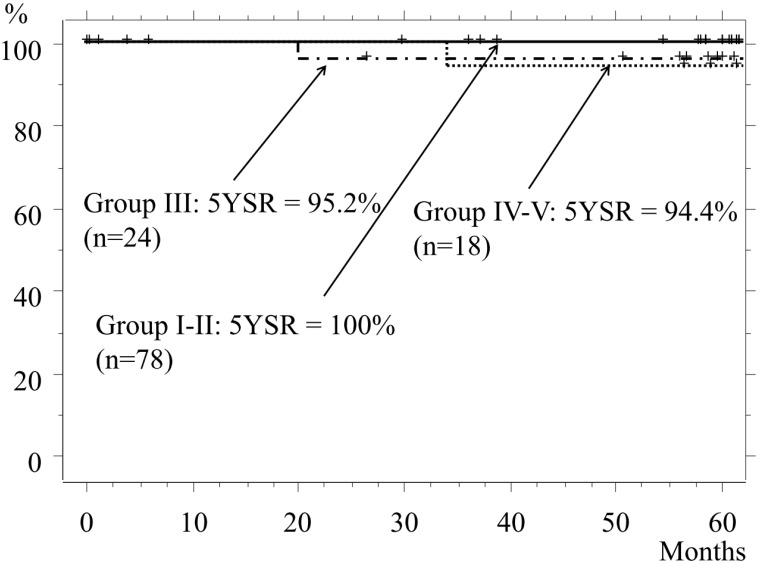
Overall survival. The 5YSR for Group I and II patients is 100%, and most of those underwent a wedge resection or segmentectomy. The 5YSR for Group III and Group IV-V is 95.8% and 94.4%, respectively (III vs. IV-V, p=0.53).

The 5-year-survival rate (5YSR) of the entire cohort was 98.2%. The 5YSR of Groups I and II, which included patients who underwent mainly a wedge resection or segmentectomy, was 100%, while the rates for Groups III and Group IV-V were 95.8% and 94.4%, respectively (p=0.53, Fig. 3). The cause of death of patients in Group III was another type of cancer or pancreatic cancer, while that in Group IV-V was the lung cancer itself.

#### Disease-free survival

**Figure 4:**
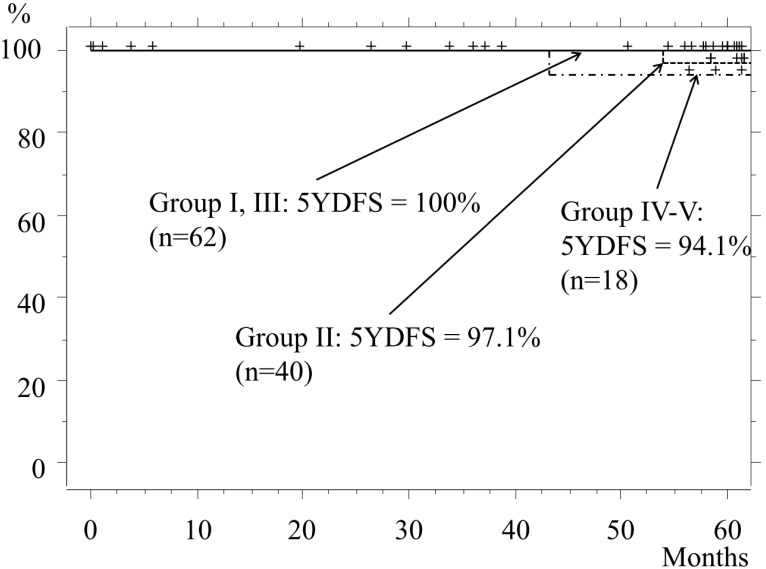
Disease-free survival. The 5YDFS rates for patients classified as Group I and Group II are 100% and 97.1%, respectively, while those in Group III and Group IV-V are 100% and 94.1%, respectively.

The 5-year disease-free survival (5YDFS) rate for the entire cohort was 98.1%, whereas the rates for Groups I and II were 100% and 97.1%, respectively. In Group II, recurrence from the margin after undergoing a wedge resection occurred in 1 patient, in whom the tumor had a GGO rate of 55% and was 11 mm in size. Even though margin cytology findings were negative, recurrence appeared at 54 months (4.5 years) after the operation, for which the patient underwent re-operation with a lobectomy. After that surgery, neither recurrence nor metastasis was seen for at least 4 years. The 5YDFS rates for Groups III and IV-V were 100% and 94.1%, respectively (Fig. 4).

## Discussion

Previous studies have reported that the malignancy of a small size pulmonary adenocarcinoma could be predicted using cytological diagnosis of imprint smears during a standard operation.^9^, ^10^ Furthermore, they concluded that such findings could provide important clinical information for planning the surgical procedure. However, there are no known reports of cytological results being applied to a surgical procedure. This is the first study of the clinical application of cytological classification in cases of small lung adenocarcinomas.

Several studies have presented the findings of limited resection for small pulmonary adenocarcinomas using intraoperative frozen sections.^5^, ^6^ However, for tumor diagnosis with a frozen section, an adequately sized sample is required, which is difficult with small tumors. On the other hand, an imprint smear method only uses the surface of the section and can provide a diagnosis within 15 minutes. In addition, we previously reported that the N-H classification is correlated with the IASLC adenocarcinoma pattern classification, the 4^th^ WHO histological grading, and the 8^th^ IASLC/ATS/ERS classification.^8^ Therefore, for intraoperative small tumor diagnosis, a cytological approach has some advantages over a frozen section method.

The indications for surgery in the present patients were scrupulously selected. Tumors that were ≤2 cm in size or >1.5 cm with less than 50% GGO were included. We previously reported that the GGO rate was related to prognosis in patients with small adenocarcinomas.^11^ In addition, another of our previous reports showed that the survival and disease-free rates of patients after a lobectomy for adenocarcinomas sized 16-20 mm and containing less than 50% GGO were significantly worse than for patients who underwent wedge resection for GGO-predominant adenocarcinomas.^12^

Cases with mucinous lesions were not included in the present study, because the smear sample from them contains mainly mucus and histiocytes, rarely tumor cells. In addition, a mucinous adenocarcinoma can easily spread in an aerogenous manner.^13^, ^14^ Furthermore, their tumor cells can spread from a macroscopic lesion widely throughout the surrounding lung parenchyma in an area larger than expected. Therefore, we considered that limited surgery was not indicated for cases with a mucinous lesion.

For preoperative diagnosis, positron emission tomography (PET) scanning has been used to diagnose a tumor's malignancy. We began to use PET scans from 2003. In the beginning, we did the scans only for selected cases. The examination has now been routinely performed since 2010. Therefore, PET scans were not available for all cases in the present cohort, but only for 57 cases. Just for information, the N-H classification correlated with the SUV-max of the PET scan (R=0.577, Supplement Figure 3). However, a PET scan can be affected not only by the tumor's malignancy, but also by its size and tissue density. In small tumors, there can often be false-negative tumors based on the SUV-max value (as a reference, see also Supplement Figure 3). This means that, if SUV-max is high, a lobectomy (or standard operation) is needed, but if the SUV-max is low, we cannot say that the tumor is non-invasive carcinoma. A low SUV-max can include also, for example, minimally invasive adenocarcinoma. Unfortunately, to determine whether a wedge resection is indicated, diagnosis by PET scan cannot be considered sufficient.

The patient in Group II with positive lymphatic invasion had no recurrence 5 years after the limited operation, while others with positive lymphatic invasion underwent a lobectomy. Lymphatic and vessel involvement was largely avoided with the limited resection of our therapeutic strategy.

The outcomes of Group I-II were acceptable even after the limited resection procedure, with acceptable disease-free survival in the Group I patients. However, 1 patient classified as Group II had margin recurrence. A detailed retrospective examination of the sample used for margin cytology showed only a few small carcinoma cells on the slide. Therefore, the difference between Group I and Group II was not caused by a difference in malignancy, but rather a limitation of the margin scanning procedure. Fortunately, that patient underwent a re-operation 4.5 years after the first operation and has since shown a good outcome.

## Conclusion

Use of cytological findings along with the N-H classification for determining operation extent is effective for patients with small pulmonary adenocarcinomas.

## Limitations

This study was conducted at a single institute, and the number of subjects, especially in Group V, was inadequate for definitive conclusions. In addition, the N-H classification is a qualitative system, though we are attempting to improve the scoring system for quantitation.

In our institute, we have used video-assisted thoracic surgery (VATS) since 2000. First, we used VATS technique only for selected cases with a small thoracotomy. Since 2010, we do complete VATS for patients with a small tumor. Because of the diverse indication for VATS and the gradual change of the procedure, it was not mentioned in this article.

Furthermore, the same samples were diagnosed and scored at multiple institutes, with the results compared.

## Supplementary Material

Supplementary figures and tables.

## Figures and Tables

**Table 1:** Nakayama-Higashiyama's classification of small pulmonary adenocarcinoma^8^

	Group I	Group II	Group III	Group IV	Group V
**Cellularity**	poor	moderate	hyper	hyper	hyper
**Size of cluster**	10-30 cells	slightly large cluster	small to large cluster	single to large cluster	single to large cluster
**Shape of cluster**	sheet-like appearance	mainly sheet-like appearance, partly overlapping	irregular overlapping	scattered isolated cells to irregular overlapping	scattered isolated cells to irregular overlapping
**Size of cells**	small	small to medium	small to large	large	large
**Dyskaryosis**	none	slight	often	often	marked
**Size of nucleus**	small & uniform size	small to medium & anisokaryosis	small to large & anisokaryosis	large & anisokaryosis	large & anisokaryosis
**Chromatin pattern**	thick, fine and granular chromatin with regular distribution	thick to sparse and fine, granular chromatin	fine granular chromatin with irregular distribution	fine granular chromatin with irregular distribution	fine to coarse, granular chromatin with irregular distribution
**Distance of inter-nucleus**	slightly irregular	slightly irregular	irregular	irregular	irregular

**Table 2:** Patients' characteristics (n=120)

Age (y, mean ± SD*)	61.6±10.1
Sex (male / female)	50 / 70
Tumor size (mm, mean ± SD)	13.1±4.1
Ground-glass opacity rate (%, mean ± SD)	70.8±30.0
Operation procedure (wedge/seg^2^* /lobectomy)	67 / 31 / 22
N1-positive status (wedge / seg /lobectomy)	unknown / 0 / 1
Lymphatic invasion (ly factor: + / -)	9 / 111
Vessel invasion (v factor: + / -)	5 / 115
Nakayama-Higashiyama's classification (Group I/II/III/IV/V)	38 / 40 / 24 / 17 / 1
IASLC/ATS/ERS classification (AI+M / L+A+P / S+MP)^2*^	62 / 47 / 2

* Standard distribution. 2* Wedge resection/segmentectomy. 3* Three cases showed atypical adenomatous hyperplasia. IASLC/ATS/ERS: International Association for the Study of Cancer (IASLC)/American Thoracic Society (ATS)/European Respiratory Society (ERS) classification; AI+M: adenocarcinoma *in situ* + minimally invasive adenocarcinoma; L+A+P: lepidic predominant adenocarcinoma + acinar predominant adenocarcinoma + papillary predominant adenocarcinoma; S+MP: solid predominant adenocarcinoma + micro-papillary predominant adenocarcinoma.

## Abbreviations

TN: Investigation, Data curation, Writing - original draft
TN: Conceptualization
TT: Data curation
AT: Investigation, Resources
HK, HI: Data curation
YT, SN, HN: Investigation, Resources
JO: Data curation
MH: Conceptualization, Data curation, Formal analysis, Methodology, Project administration, Writing - review & editing
