# Supplementary figures and images for: Intraoperative Diagnosis and Surgical Procedure with Imprint Cytology for Small Pulmonary Adenocarcinoma
Source: J Cancer. 2020 Feb 20;11(10):2724–9. doi: 10.7150/jca.35026 (PMC7086261; doi:10.7150/jca.35026)

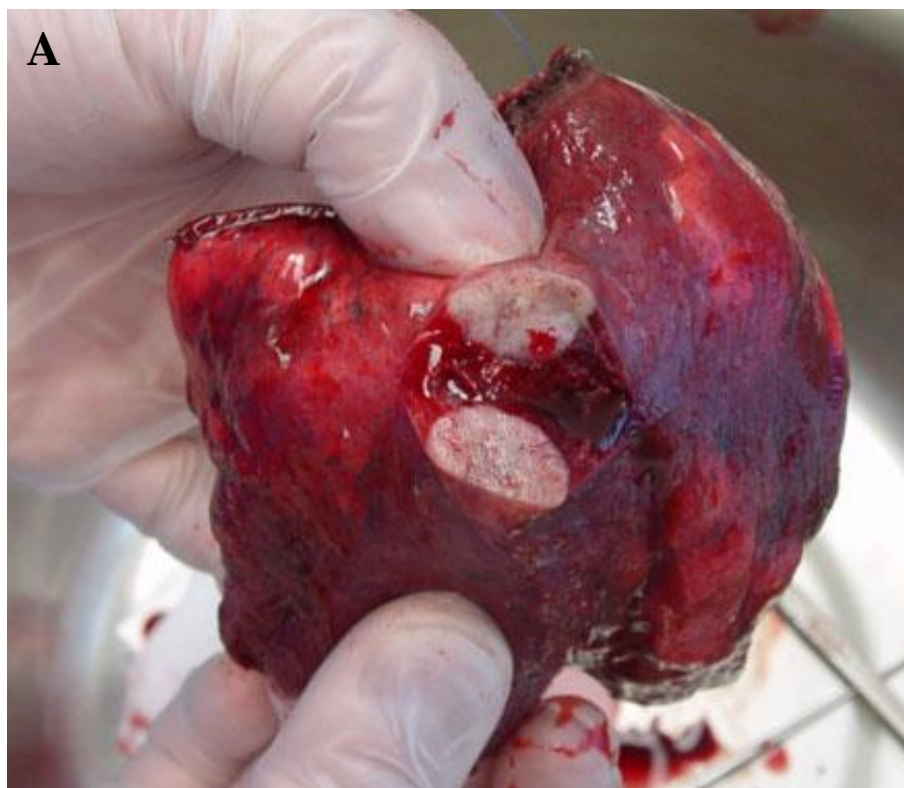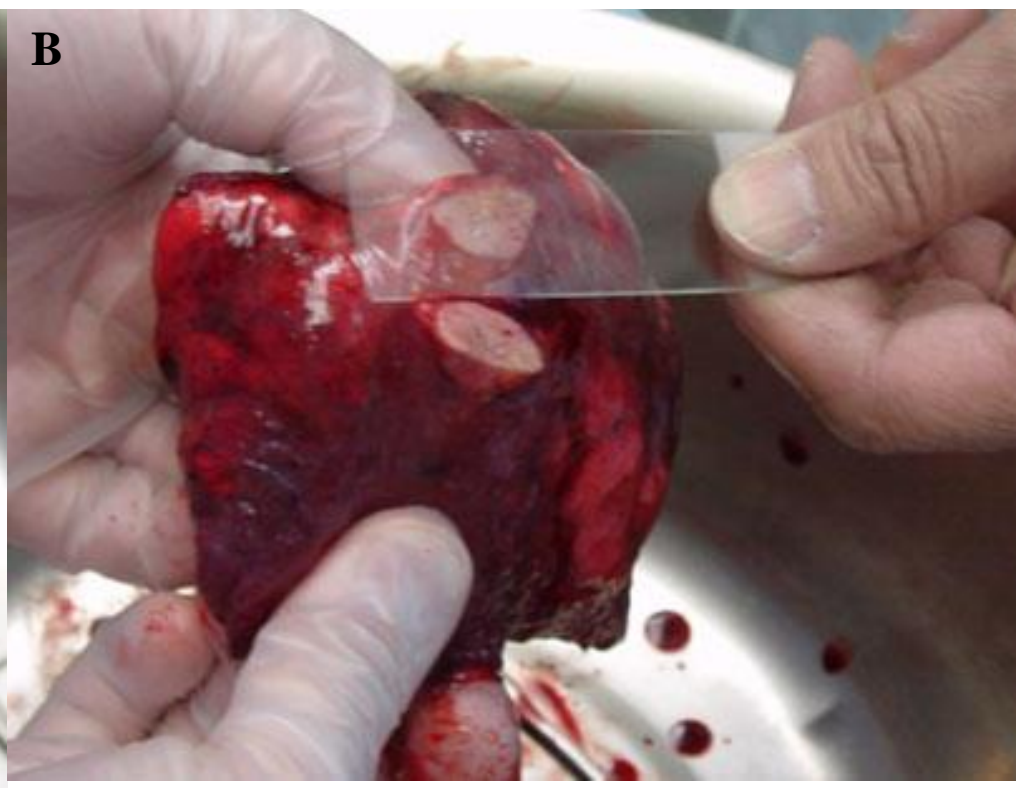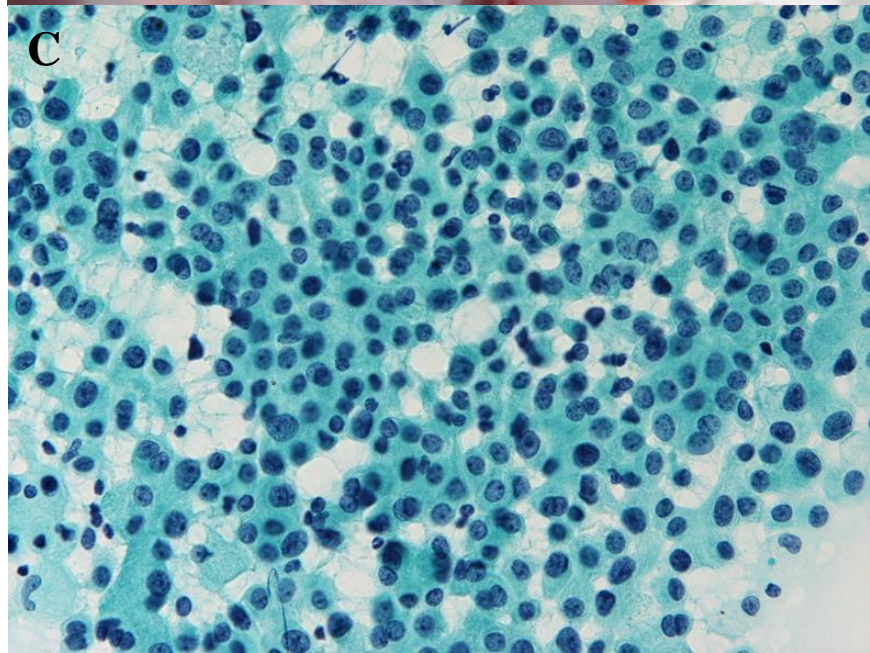

# I

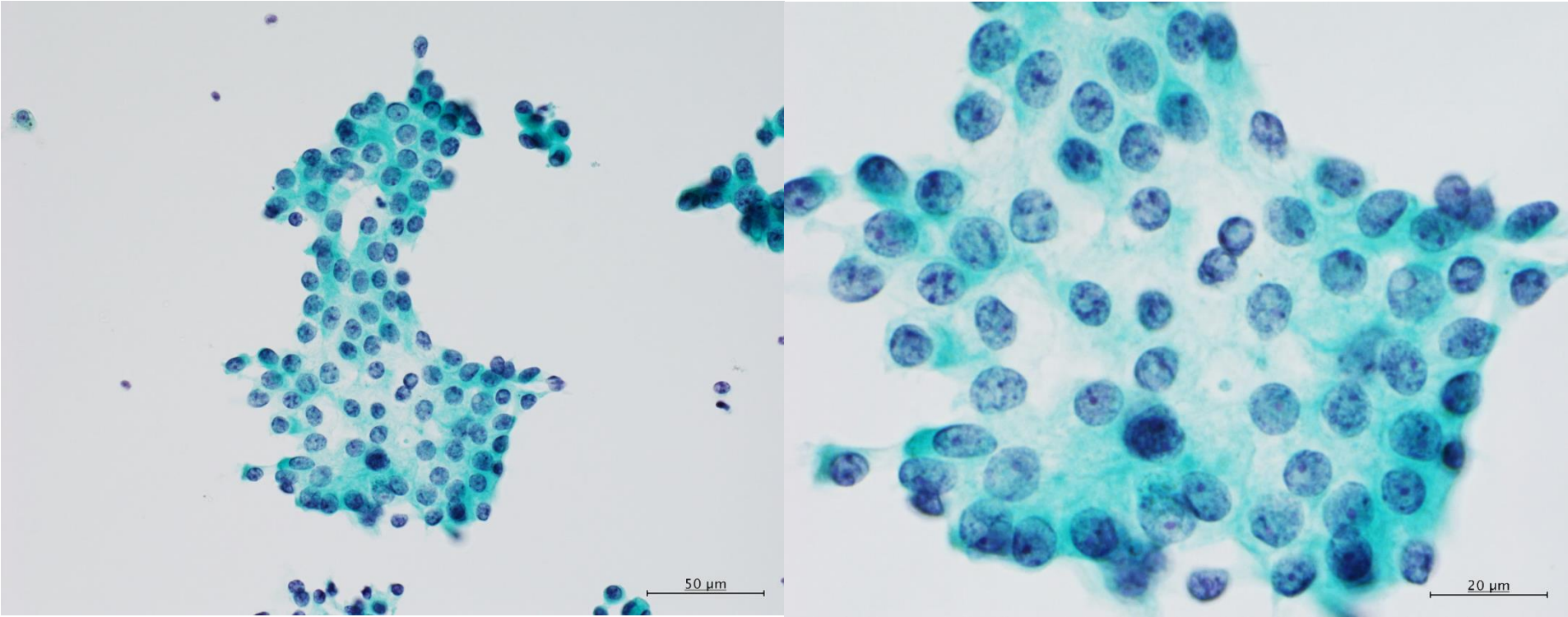

# II

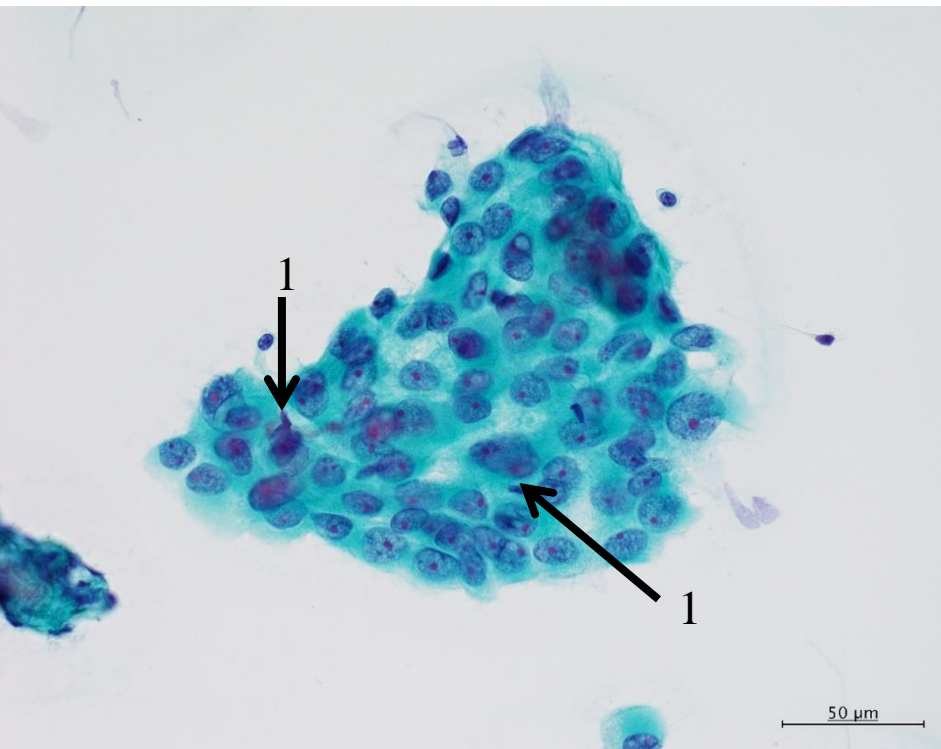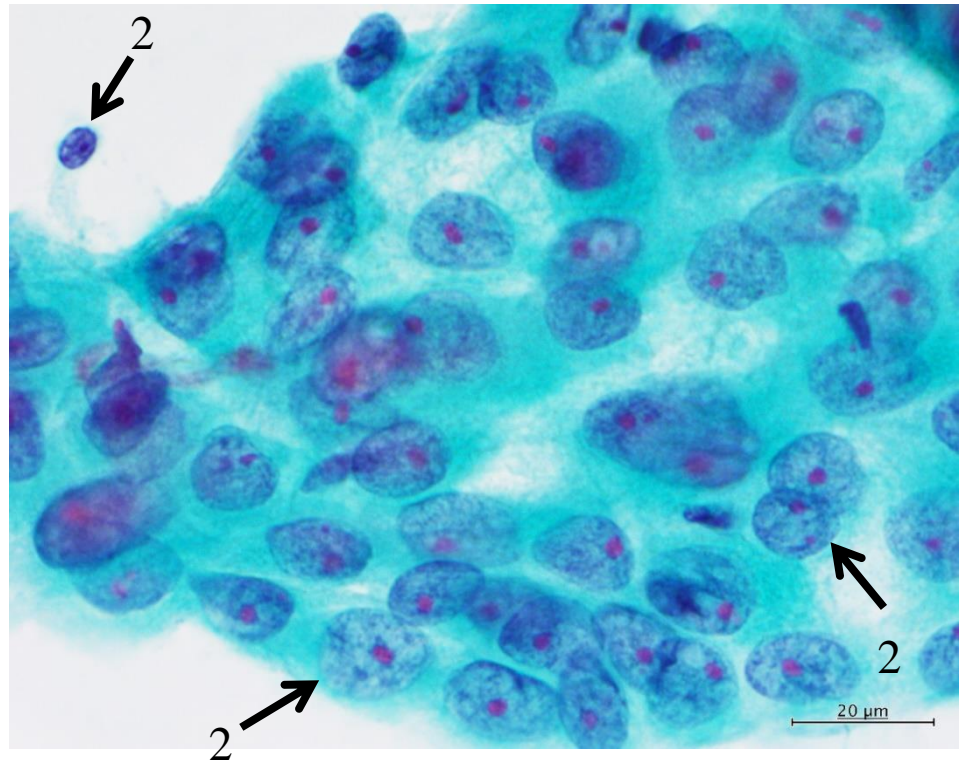

# III

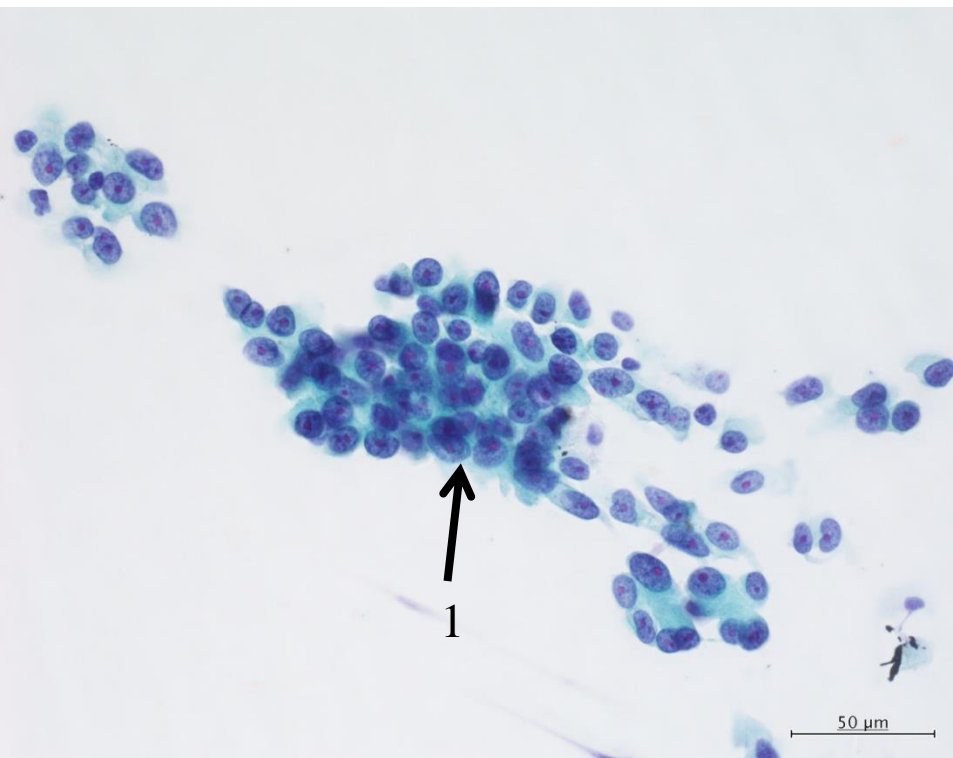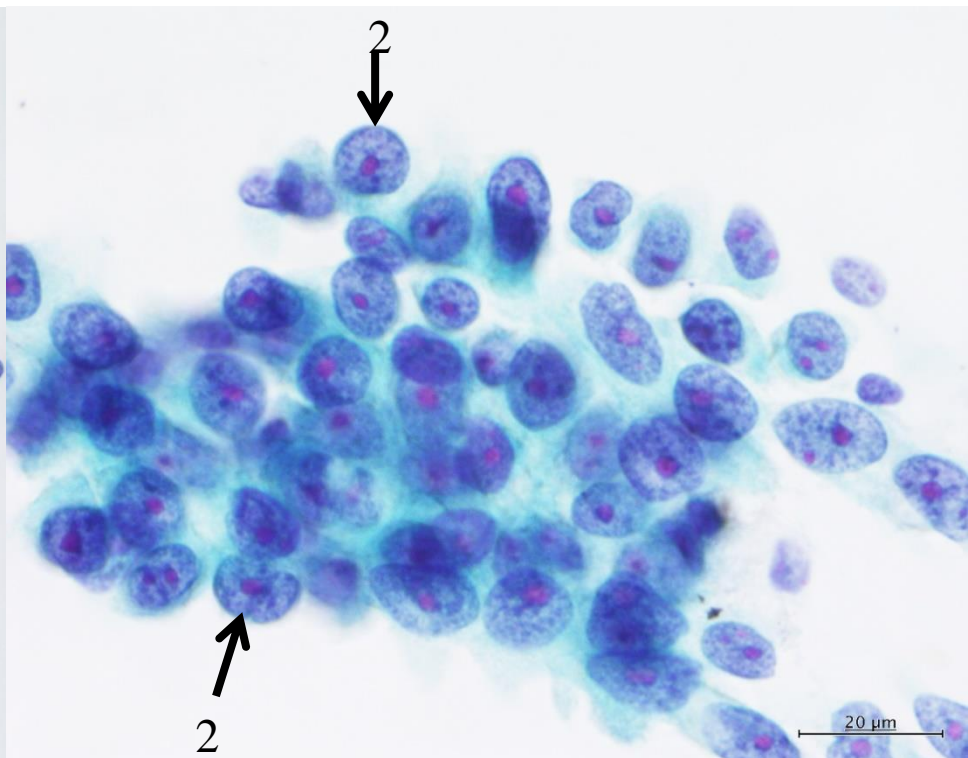

# IV

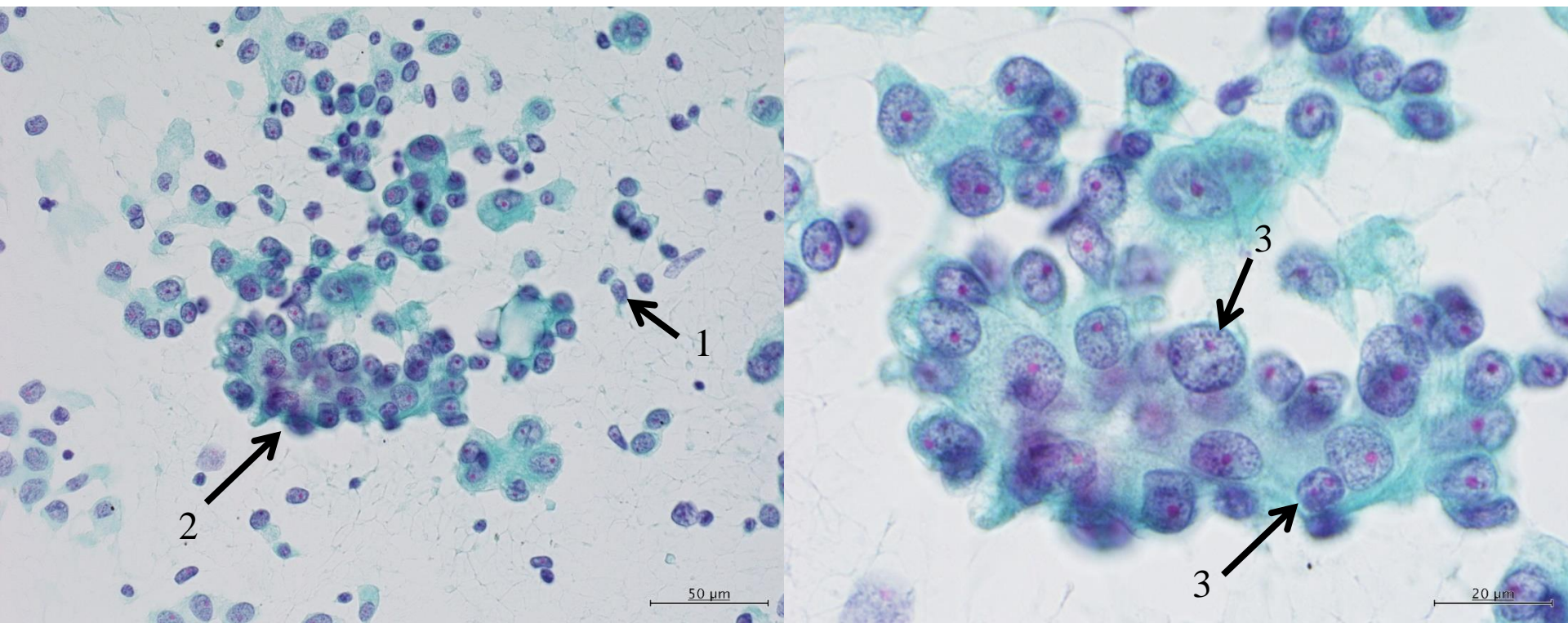

V

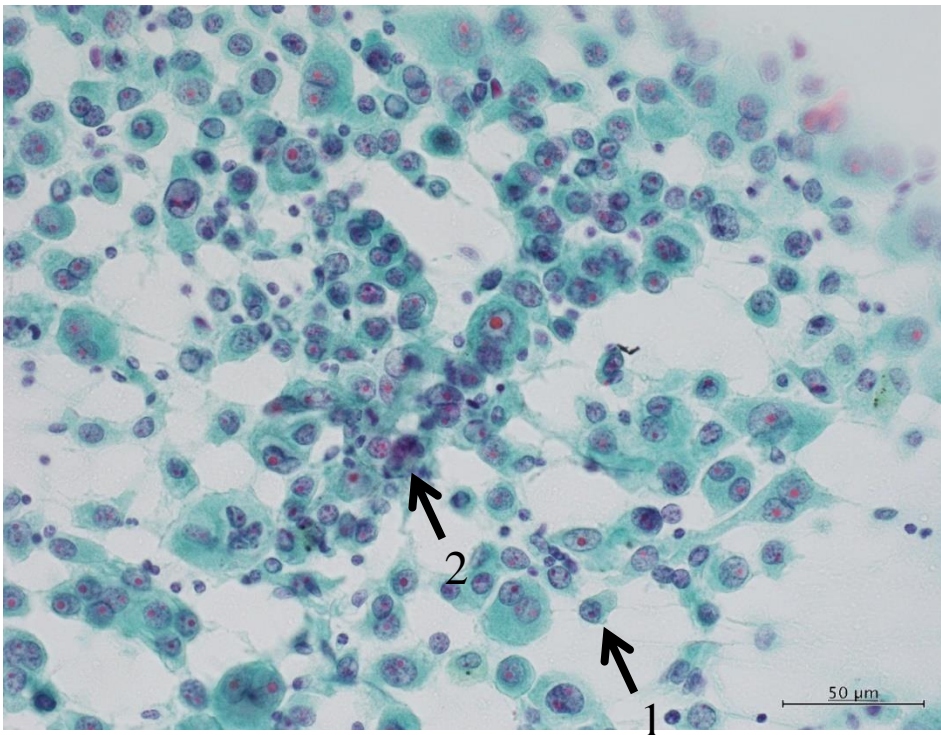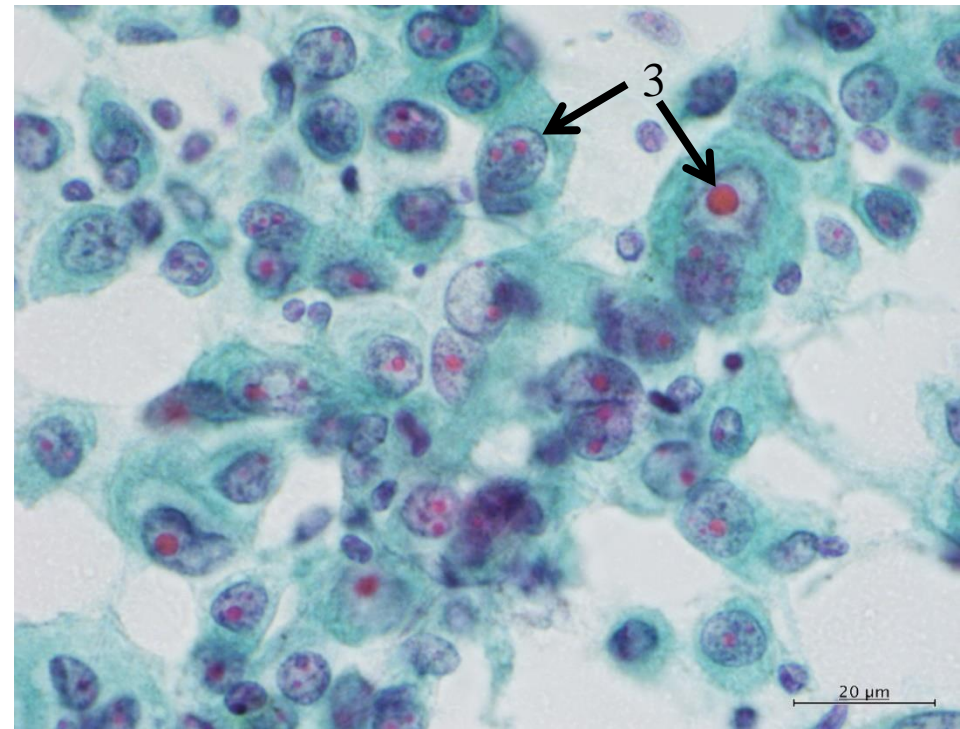

Supplement figure 3

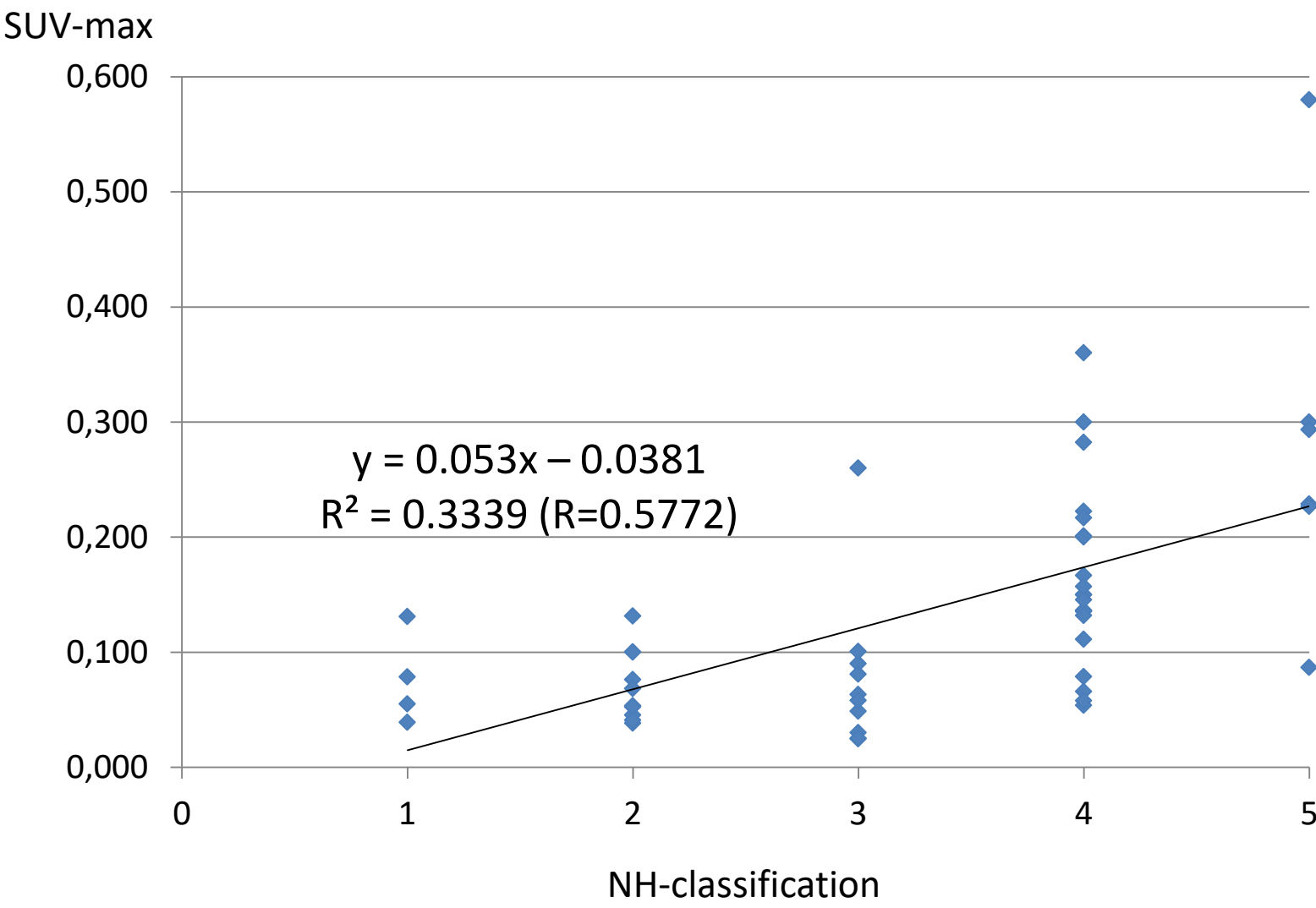

Supplement: Supplementary file 1 — Supplementary figures and tables. [file jcav11p2724s1.pdf]
